# A Novel Dual-Ion Capacitive Deionization System Design with Ultrahigh Desalination Performance

**DOI:** 10.3390/polym14214776

**Published:** 2022-11-07

**Authors:** Yuxin Jiang, Zhiguo Hou, Lvji Yan, Haiyin Gang, Haiying Wang, Liyuan Chai

**Affiliations:** 1 School of Metallurgy and Environment, Central South University, Changsha 410083, China; 2 School of Chemistry and Materials, University of Science and Technology of China, Hefei 230026, China; 3 Chinese National Engineering Research Center for Control and Treatment of Heavy Metal Pollution, Changsha 410083, China; 4 Water Pollution Control Technology Key Lab. of Hunan Province, Changsha 410083, China

**Keywords:** capacitive deionization, desalination, dual-ion, battery electrode

## Abstract

Capacitive deionization is an emerging desalination technology with mild operation conditions and high energy efficiency. However, its application is limited due to the low deionization capacity of traditional capacitive electrodes. Herein, we report a novel dual-ion capacitive deionization system with a lithium-ion battery cathode LiMn_2_O_4_/C and a sodium-ion battery anode NaTi_2_(PO_4_)_3_/C. Lithium ions could enhance the charge transfer during CDI desalination, while NaTi_2_(PO_4_)_3_/C provided direct intercalation sites for sodium ions. The electrochemical capacities of the battery electrodes fitted well, which was favorable for the optimization of the desalination capacity. The low potential of the redox couple Ti^3+^/Ti^4+^ (−0.8 V versus Ag/AgCl) and intercalation/deintercalation behaviors of sodium ions that suppressed hydrogen evolution could enlarge the voltage window of the CDI process to 1.8 V. The novel CDI cell achieved an ultrahigh desalination capacity of 140.03 mg·g^−1^ at 1.8 V with an initial salinity of 20 mM, revealing a new direction for the CDI performance enhancement.

## 1. Introduction

Desalination is an inevitable choice for solving the global freshwater scarcity crisis, which has been a hotspot of scientific research over recent years [1,2,3]. A number of techniques have been put into industrial application, such as multi-effect distillation, multi-stage flash, membrane distillation, reverse osmosis, nano/ultrafiltration, etc. [4,5,6,7,8,9]. However, these methods are mainly thermal-driven or pressure-driven, which are energy-intensive. On the other hand, capacitive-deionization (CDI), as an emerging energy-efficient desalination technology that employs electrode materials to adsorb salt from saline water, could operate under mild conditions [10,11]. Moreover, energy could be stored simultaneously during desalination in CDI [12], which is a mechanism similar to a capacitor [13]. 

Yet, the CDI technology has already been scaled up by only a few commercial companies (EST Water and Technologies in China, Current Water Technologies in Canada, etc.) [14]. The main obstacle hindering the wide application of CDI is the low desalination capacity [15]. Traditional electrode materials in CDI are mainly carbon-based materials. These materials adsorb salt with the mechanism of electric double layers; thus, the capacity is strongly limited by the specific surface area of the carbonaceous material (<15 mg·g^−1^) [16,17]. Nevertheless, battery electrode materials with larger electrochemical capacities, which could store salt ions not only on the surfaces but also in the inner crystal structures, have shown great potential in CDI desalination [18,19]. Numerous battery electrodes have been employed in CDI to obtain high desalination capacities. Lee [20] used a Na_4_Mn_9_O_18_ electrode in a hybrid CDI (HCDI) system with activated carbon (AC) as the counter electrode, and a deionization capacity of 31.2 mg·g^−1^ was achieved. Ahn [21] coupled chloride-capture electrode Ag with another battery electrode AgCl, and reached a high desalination capacity of 85 mg·g^−1^. Furthermore, in order to enhance the charge transfer in the CDI processes, carbon-based materials are usually incorporated into battery electrode materials. For example, Yue [22] employed Na_4_Ti_9_O_20_/C in an HCDI cell and reached a CDI capacity of 66.14 mg·g^−1^, which was nearly twice that with the pure Na_4_Ti_9_O_20_ electrode. Moreover, it is noticeable that most of the battery electrodes utilized in CDI are mainly sodium-ion batteries or chloride-ion battery electrodes, owing to the direct relationship with the composition of the salt. Theoretically, the lithium-ion could be a more efficient charge carrier compared to these salt ions because of the smaller mass per unit of carried charge [23,24,25,26], and better desalination performance might be brought out. Based on this consideration, our group synthesized lithium-ion battery material LiMn_2_O_4_/C, which was further employed as the cathode in an HCDI device [27]. The CDI cell exhibited a superior desalination capacity of 117.3 mg·g^−1^ compared to other deionization performances. However, the anode material in this HCDI cell was AC, the capacity of which was far lower than that of the cathode, restricting the further enhancement of the desalination capacity of the system.

Herein, we present a novel dual-ion CDI system with a lithium-ion battery cathode and a sodium-ion battery anode. The sodium superion conductor (NASICON) material NaTi_2_(PO_4_)_3_/C was chosen as the anode material. NaTi_2_(PO_4_)_3_ is a stable aqueous sodium-ion anode material that could capture sodium ions during CDI [28], and the coating of carbon could facilitate the charge transfer within the process [29]. The reversible capacity of NaTi_2_(PO_4_)_3_/C was tested to be 98.63 mAh·g^−1^, and beneficial for the full exploitation of the cathode capacity (70.57 mAh·g^−1^). The simultaneous redox behaviors of the electrodes were studied with characterization methods. The dual-ion cell achieved an ultrahigh desalination capacity of 140.03 mg·g^−1^ with an initial salt concentration of 20 mM, which could open up a new window for the elevation of CDI desalination efficiency.

## 2. Materials and Methods

### 2.1. Experimental Agents

Spinel LiMn_2_O_4_ (LMO) was purchased from Ziyi Co., Ltd. (Shanghai, China). Anion-exchange membrane (AMX) was purchased from ASTOM Corp. (Tokyo, Japan). Polyvinylidene fluoride (PVDF, HSV900) was purchased from MTI Corp. (Richmond, CA, USA). Carbon black was purchased from Cabot Corp. (Boston, MA, USA). N-Methylpyrrolidone (NMP, 99%) and polytetrafluoroethylene preparation (PTFE, 60%) was purchased from Aladdin Corp. NaCl (≥99.8%), LiCl (analytical grade) and glucose (analytical grade) were purchased from Sinopharm Group. (Beijing, China). NaH_2_PO_4_ (99%), NH_4_H_2_PO_4_ (99%), and TiO_2_ (99%) were purchased from Macklin Co., Ltd. (Shanghai, China). Hydrophilic carbon paper was purchased from TORAY Industries, Inc., titanium plates were purchased from Rulin Co, Ltd. (Changsha, China), and titanium mesh was purchased from Kangwei Co., Ltd. (Hengshui, China). AC was purchased from Nanjing XFNANO Materials Tech Co., Ltd. (Nanjing, China).

### 2.2. Preparatrion of Materials

The LiMn_2_O_4_/C composites were prepared with a simple ball-milling method according to our previous study [25], spinel LMO and carbon black were mixed with a mass ratio of 2:1 and then milled in a ball mill (YXQM-4L, MITR) for 6 h.

NaTi_2_(PO_4_)_3_/C was synthesized by a hydrothermal–spraying–calcination method [30]. Stoichiometric amounts of NaH_2_PO_4_, NH_4_H_2_PO_4_, and TiO_2_ were used in a hydrothermal process at 150 °C to produce the precursor of the material. Glucose was then mixed with the precursor and the composites were sprayed and annealed at 700 °C in N_2_ afterwards.

### 2.3. Characterization

Field-emission scanning electron microscope (FESEM, JSM-7900F, JEOL) and transmission electron microscope (TEM, Titan G2 60-300 with image corrector, FEI) were used to observe the structures of the materials. Energy dispersion spectroscopy (EDS, Octane Elect Super, EDAX) was used to study the element dispersion of the material. X-ray diffraction (XRD, Empyrean 2, PANalytical) and X-ray photoelectron spectroscopy (XPS) were used to obtain information on structures and valences in the composites. Infrared carbon and sulfur analyzer (CS844, LECO) was used to measure the carbon content in the prepared material. The Brunauer−Emmett−Teller (BET) tests were carried out with the surface area and pore size analyzer (KUBOX1000, Bjbuilder).

### 2.4. Fabrication of Electrodes

As for the fabrication of LiMn_2_O_4_/C electrode or AC electrode, active material, carbon black, and PVDF were ground with a mass ratio of 8:1:1 to obtain a homogeneous mixture that was further mixed with NMP for another 5 min to form a slurry. It was then painted onto the surface of carbon paper for the electrochemical test or titanium plate for the CDI test. Afterwards, the current collectors were kept at the temperature of 90 °C for 4 h to complete the electrode preparation.

The fabrication of the NaTi_2_(PO_4_)_3_/C electrode was basically similar to that of the LiMn_2_O_4_/C electrode, however, there were some differences. The binder used in the preparation of NaTi_2_(PO_4_)_3_/C electrode was PTFE. The mass ratio of active material, carbon black, and binder was 9:0.5:0.5. Moreover, when preparing the NaTi2(PO4)3/C electrode, titanium mesh, rather than carbon paper, was used as the current collector. A rolling machine (MSK-2150, KEJING) was used to compress the slurry on the mesh before the heat treatment.

### 2.5. Electrochemical and Deionization Tests

All the electrochemical and desalination tests were conducted with the electrochemical workstation (Multi autolab/M204, Metrohm). Galvanostatic charge–discharge (GCD) tests were used to measure the specific capacities of the electrode materials according to Equation (1):

(1) $$C_{s}=\frac{I\times t}{3.6\times m},$$

in which *C_s_* (mAh·g^−1^) is the specific capacity of the material, *I* (A) refers to the constant current intensity, *t* (s) denotes the discharging time in the test, and *m* (g) represents the mass of the active material in the electrode. Besides, the corresponding coulombic efficiency (η) was calculated as follows:

(2) $$\eta =\frac{C_{charge}}{C_{discharge}}$$

where *C_charge_* is the charge capacity and *C_discharge_* stands for the discharge capacity. Furthermore, the redox behaviors of the electrode materials were investigated with the cyclic voltammetry method in a 3-electrode system with Ag/AgCl as the reference electrode and platinum as the counter electrode, which was also employed in the GCD test to measure the electrochemical capacity of certain electrode material. In addition, the salt removal capacity (*SAC*, mg·g^−1^) in the desalination test was calculated as Equation (3):

(3) $$SAC=\frac{\left(C_{f-}C_{0}\right)\times V}{M},$$

in which *C_f_* (mg·L^−1^) and *C_0_* (mg·L^−1^) are the final and initial salt concentrations of the feed solution during the deionization test, *V* (L) represents the volume of the saline solution, and *M* (g) stands for the total mass of the active materials in the CDI electrodes. The concentrations of chloride ions were measured with ion chromatography (883 Basic IC Plus, Metrohm) and the CDI device was charged with the electrochemical workstation. Moreover, the salt removal rate (*SAR*, mg·g^−1^·min^−1^) was calculated as follows:

(4) $$SAR=\frac{SAC}{T}$$

where *T* denotes the CDI reaction time (min). Additionally, the pH variations during the desalination tests were measured with a pH meter ((PHSJ-3F, Leica).

## 3. Results and Discussion

### 3.1. Characterization

**Figure 1:**
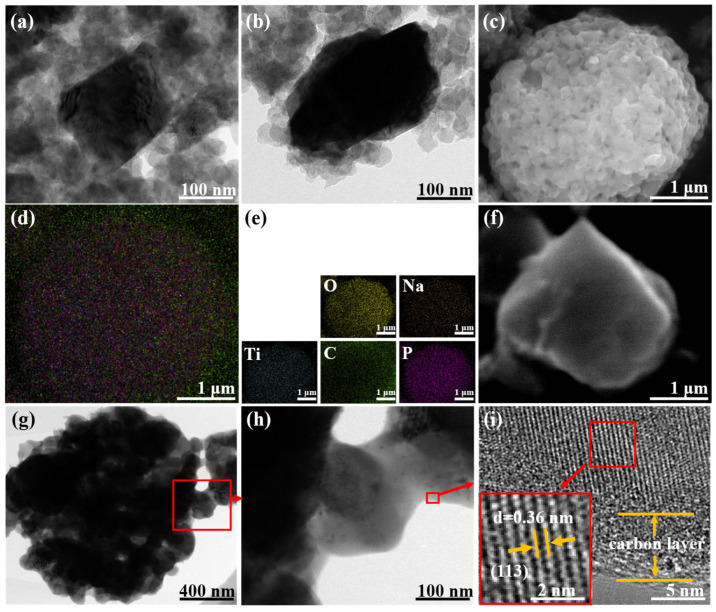
(**a**,**b**) TEM images of LiMn_2_O_4_/C material; (**c**,**f**) SEM and (**d**,**e**) EDS images of NaTi_2_(PO_4_)_3_/C material; (**g**–**i**) TEM images of NaTi_2_(PO_4_)_3_/C.

The structure of LiMn_2_O_4_/C is shown in Figure 1a,b. The nano-sized spinel LMO particles were surrounded by carbon spheres, which enhanced the charge transfer. Other structural information on LiMn_2_O_4_/C could be acquired in our published article [25]. As shown in Figure 1c–f, rectangular NaTi_2_(PO_4_)_3_/C particles (200–300 nm) are agglomerated and the elements are homogeneously dispersed. The TEM images (Figure 1g–i) show clearer crystal structures of the NaTi_2_(PO_4_)_3_/C particle and the obvious carbon coating layer of about 5 nm, which could offer a charge transfer facility and crystal protection. According to the infrared carbon and sulfur analyzer, NaTi_2_(PO_4_)_3_/C had a carbon content of 3.11%.

**Figure 2:**
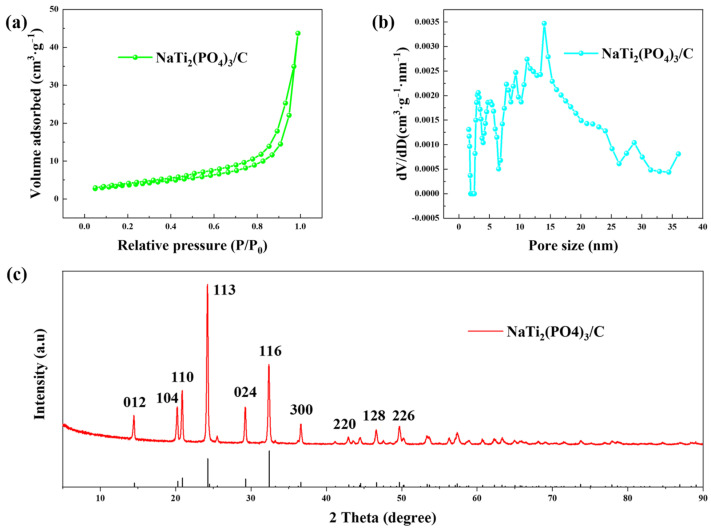
The (**a**) N_2_ adsorption-desorption isotherm, (**b**) pore size distribution, and (**c**) XRD pattern of NaTi_2_(PO_4_)_3_/C.

The BET results of prepared NaTi_2_(PO_4_)_3_/C are shown in Figure 2a,b. The material had a specific surface area of 10.23 m^2^·g^−1^, and the pore sizes were mainly distributed in the range of 5~30 nm. The XRD pattern of the anode material is presented in Figure 2c, which fits well with the standard pattern (JCPDS NO.33-1296), implying the successful synthesis of NaTi_2_(PO_4_)_3_/C.

### 3.2. Electrochemical Tests

In the hybrid CDI system, AC is the most employed counter electrode of the battery electrode [31,32,33]. In this study, we employed the NaTi_2_(PO_4_)_3_/C composites with the sodium ion intercalation behavior to replace AC. Electrochemical tests were carried out to study the potential differences between the two materials as CDI electrodes.

**Figure 3:**
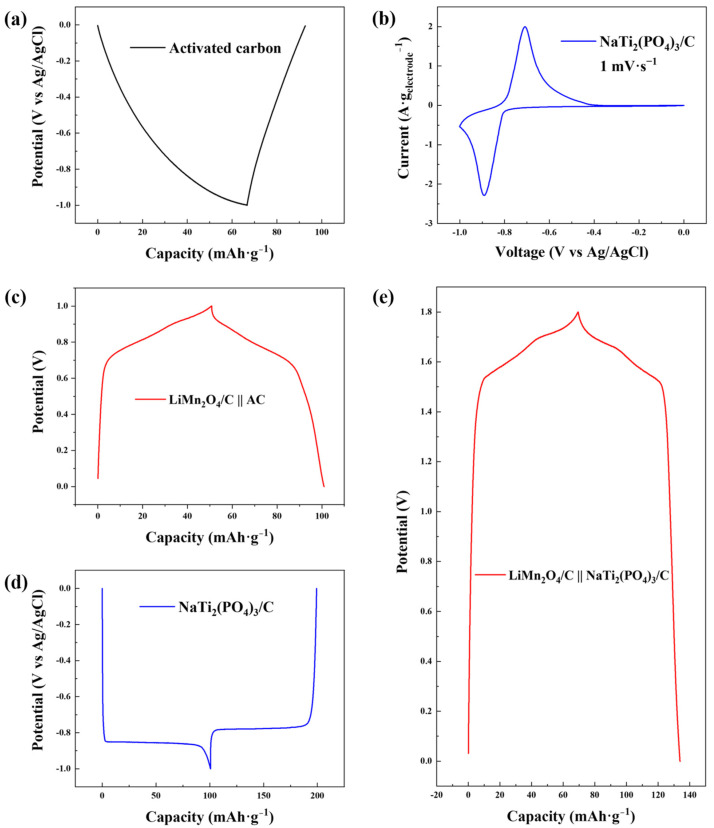
(**a**) GCD profile of AC in 1.0 M NaCl solution with the current density of −0.1 A·g^−1^ with Ag/AgCl as the reference electrode; (**b**) cyclic voltammetry curve of NaTi_2_(PO_4_)_3_/C in 1.0 M NaCl solution with the scan rate of 1 mV·s^−1^; (**c**) GCD profile of LiMn_2_O_4_/C in 1.0 M LiCl solution at the current density of 0.1 A·g^−1^ with AC as the counter electrode in the two-electrode system; (**d**) GCD profile of NaTi_2_(PO_4_)_3_/C in 1.0 M NaCl solution at the current density of −0.1 A·g^−1^ with Ag/AgCl as the reference electrode; (**e**) GCD profile of LiMn_2_O_4_/C in a mixed solution (0.5 M LiCl + 0.5 M NaCl) at the current density of 0.1 A·g^−1^ with NaTi_2_(PO_4_)_3_/C as the counter electrode in the two-electrode system.

As shown in Figure 3a, the electrochemical plateaus of the AC electrode during the GCD test with a low voltage range (−1.0~0 V versus Ag/AgCl) were evidently asymmetrical, indicating severe hydrogen evolution, which was also verified by the cyclic voltammetry curve (Figure S1a). Nevertheless, hydrogen evolution was suppressed for NaTi_2_(PO_4_)_3_/C as reversible charging and discharging plateaus were observed in the GCD profile (Figure 3d), which was due to the intercalation/deintercalation reactions of sodium ions [34]. The cyclic voltammetry curve of NaTi_2_(PO_4_)_3_/C in Figure 3b exhibits reversible redox peaks signifying the release and capture of sodium ions, and no obvious hydrogen evolution peak was observed within the scan range, further indicating the alleviation of hydrogen evolution.

Reversible charging and discharging behaviors of NaTi_2_(PO_4_)_3_/C are clear in the GCD profile and a specific electrochemical capacity of 98.63 mAh·g^−1^ was performed, 272.61% higher than that of AC (26.47 mAh·g^−1^, Figure S1b). The high capacity of the NaTi_2_(PO_4_)_3_/C anode was favorable for the full use of the capacity of the LiMn_2_O_4_/C cathode (70.57 mAh·g^−1^, Figure S2a) in CDI desalination. On the other hand, as the anode acted as the counter electrode to the LiMn_2_O_4_/C cathode and the reference electrode simultaneously in CDI, a GCD test of the LiMn_2_O_4_/C-NaTi_2_(PO_4_)_3_/C couple was performed (Figure 3e). The GCD curve of the novel dual-ion electrode couple has a larger voltage window of 1.8 V, compared to that of the GCD curve in the two-electrode system with AC as the anode (1.0 V, Figure 3c). This was owing to the suppression of the hydrogen evolution reaction and the low potential of Ti^4+^/Ti^3+^ (−0.8 V versus Ag/AgCl). The GCD test of LiMn_2_O_4_/C-NaTi_2_(PO_4_)_3_/C was performed in a LiCl/NaCl mixed solution with 0.5 M as the concentrations of both solvents. No ion-exchange membrane was introduced into the two-electrode cell for the convenience of comparison with GCD tests with other electrodes. Additionally, the sodium ion could rarely be intercalated into LiMn_2_O_4_/C (Figure S2b) due to the larger ion radius (0.102 nm) compared to that of the lithium ion (0.076 nm) [35,36]. Hence, the mixed solution was chosen as the electrolyte in the capacity test of the novel electrode couple. Though the sodium ions seemed to be worse charge carriers in the GCD test than the lithium ions [37,38], the capacity of the LiMn_2_O_4_/C-NaTi_2_(PO_4_)_3_/C system with mixed electrolyte (64.43 mAh·g^−1^) was 28.37% higher than that of the LiMn_2_O_4_/C-AC system with pure LiCl electrolyte (50.19 mAh·g^−1^), revealing a robust potential of enhancing desalination performance.

### 3.3. Desalination Performance

**Figure 4:**
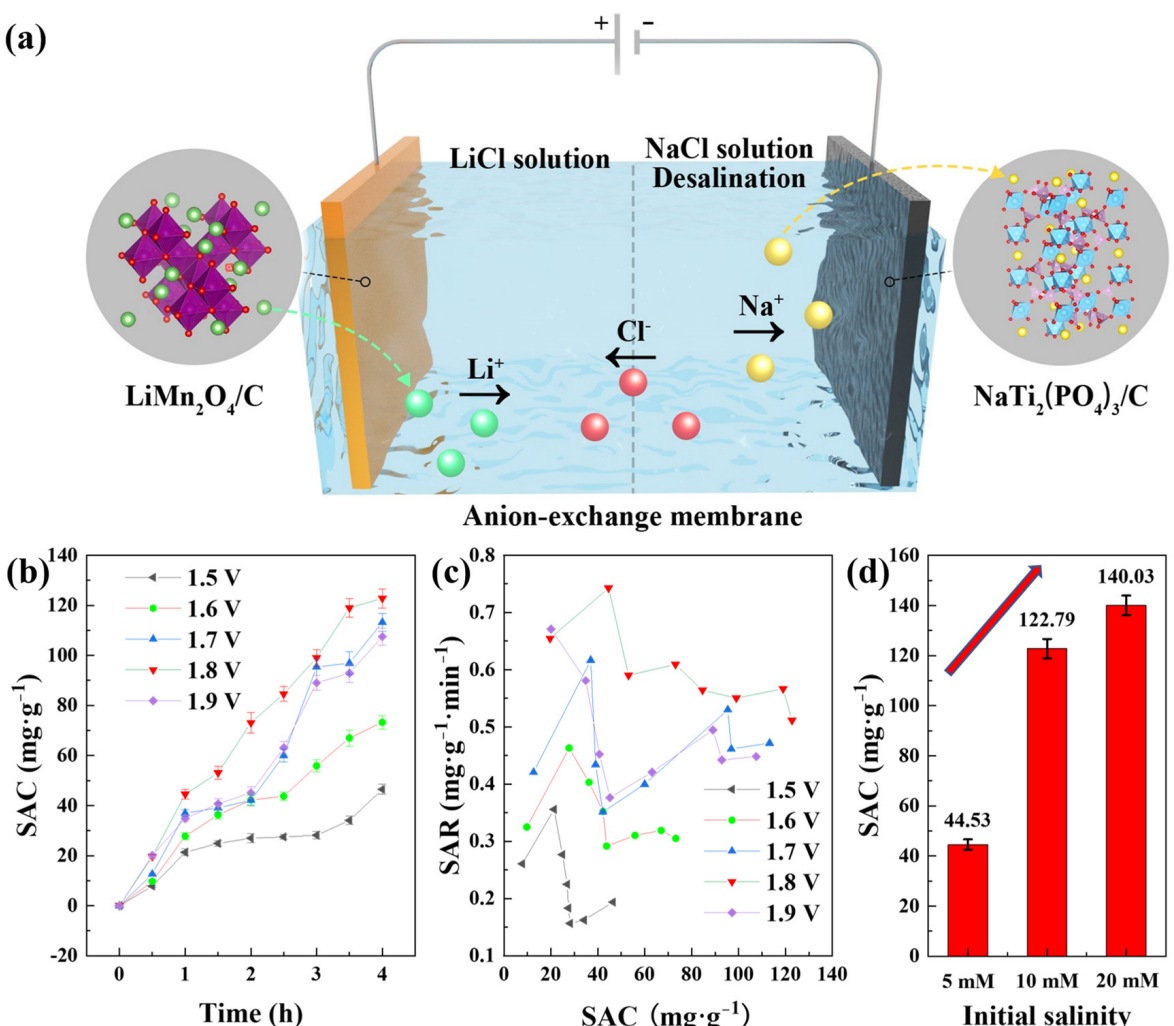
(**a**) The configuration of the CDI cell with dual-ion battery electrodes; (**b**) the deionization capacities of different voltages in the dual-ion desalination cell with the initial salinity of 10 mM; (**c**) the corresponding salt removal rates; (**d**) the desalination capacities with different initial salt concentrations.

The desalination test was conducted with the mode of constant voltage (CV) at room temperature in a CDI device, as shown in Figure 4a. The cell was divided by an anion-exchange membrane into two regions: the cathodic region with LiMn_2_O_4_/C composites as the cathode and 10 mM LiCl solution (100 mL) as the catholyte, and the anodic region with NaTi_2_(PO_4_)_3_/C composites as the anode and NaCl solution (125 mL) as the feed solution. In addition, the total mass of the active materials in the cell was approximately 100 mg, with a mass ratio of about 1:1 for the cathode and anode. When a voltage was applied to the cathode, the lithium ions could be released from LiMn_2_O_4_/C and attract the chloride ions from the other side of the membrane, and the sodium ions could be intercalated into NaTi_2_(PO_4_)_3_/C simultaneously, finishing the desalination of the feed solution.

Owing to the inhibition of hydrogen evolution at the NaTi_2_(PO_4_)_3_/C electrode, the cell could be applied by a high voltage during CDI desalination. The device was charged with different voltages and the reaction time was 4 h with an initial salt concentration of 10 mM. The corresponding desalination performances are shown in Figure 4b, from which we could figure out that 1.8 V was the appropriate voltage for high desalination performance with a deionization capacity of 122.79 mg·g^−1^. Basically, the salt removal capacity rose as the voltage increased, and 1.5 V brought out the lowest desalination capacity of 46.51 mg·g^−1^. This was owing to the fact that the higher voltage could provide a stronger electric field that facilitated ion transfer. However, it was also interesting that 1.8 V instead of 1.9 V was the applied voltage with the highest desalination performance at 10 mM, which was probably due to that more side reactions occurred at 1.9 V. The CDI cell was a two-electrode system with NaTi_2_(PO_4_)_3_/C electrode as the reference electrode, and the potential of the redox couple Ti^4+^/Ti^3+^ was about 0.8 V lower than the Ag/AgCl electrode, as shown in Figure 3d. Therefore, the voltage 1.9 V denotes an approximately 1.1 V versus Ag/AgCl applied to the LiMn_2_O_4_/C electrode. In addition, the Ag/AgCl electrode potential is approximately 0.2 V higher than the standard hydrogen electrode, revealing that the 1.9 V applied to the desalination device was close to a voltage that was 0.1 V higher than the theoretical oxygen evolution potential. Thus, less oxygen evolution was performed at the applied voltage of 1.8 V, and a higher desalination efficiency could be obtained. Furthermore, the pH values in the solutions during desalination remained steady. Figure 4c depicts the salt removal rates at different voltages, and the voltage of 1.8 V was beneficial for fast desalination. The desalination rate of the whole CDI process in the dual-ion system reached 0.51 mg·g^−1^·min^−1^, over seven times that of the HCDI cell with an AC anode [25].

The deionization capacities with different initial feed salinities at 1.8 V are shown in Figure 4d. An ultrahigh desalination capacity of 140.03 mg·g^−1^ was achieved with the initial salt concentration of 20 mM. The deionization performance was higher as the salt concentration went higher, which was likely because higher salinity offered higher conductivity and more ions for electrochemical capture. The salt removal capacities of other electrode couples in the literature are listed in Table 1, with which a clear comparison of desalination performances is revealed.

**Figure 5:**
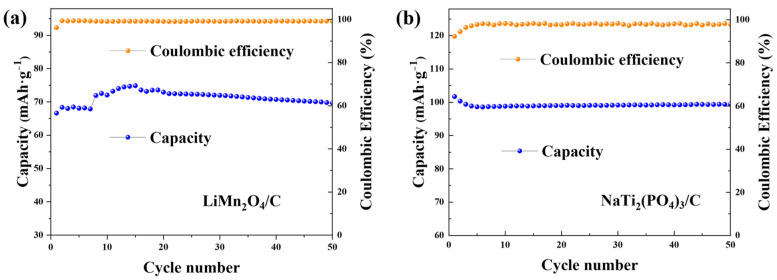
The cycling profiles of (**a**) LiMn_2_O_4_/C in 1.0 M LiCl solution and (**b**) NaTi_2_(PO_4_)_3_/C with the constant current density of 0.1 g·A^−1^.

The cycling performances of both electrodes in the CDI cell are presented in Figure 5. The capacities kept stable over the cycles, indicating the good cycling ability of the deionization device.

**Figure 6:**
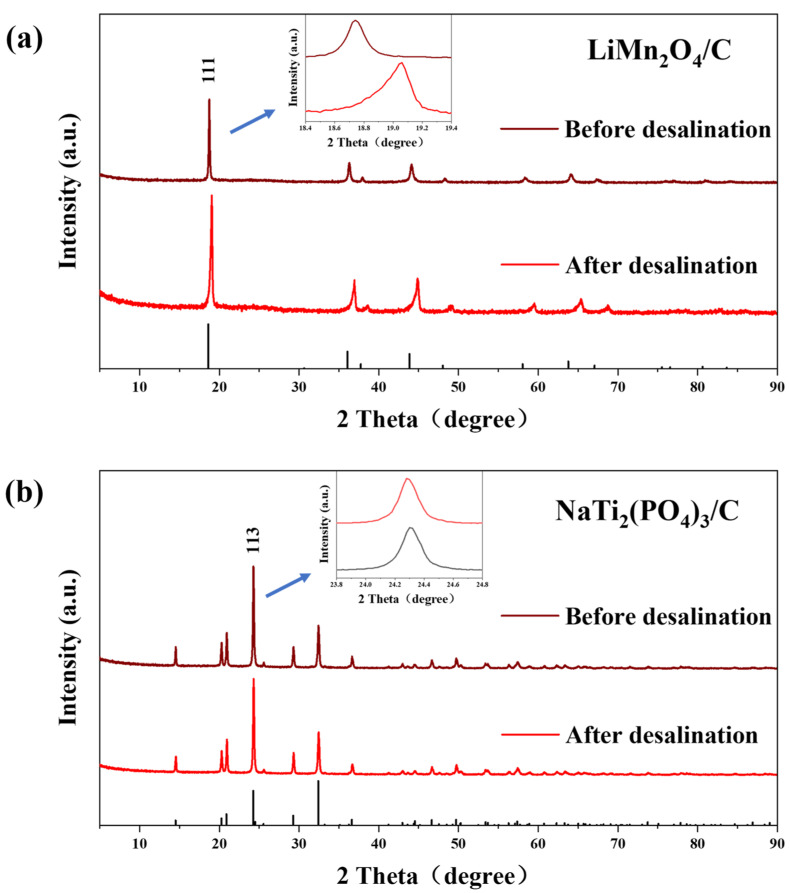
The (**a**) XRD patterns of LiMn_2_O_4_/C and (**b**) NaTi_2_(PO_4_)_3_/C before and after the desalination test at 1.8 V in 10 mM salt solution.

**Figure 7:**
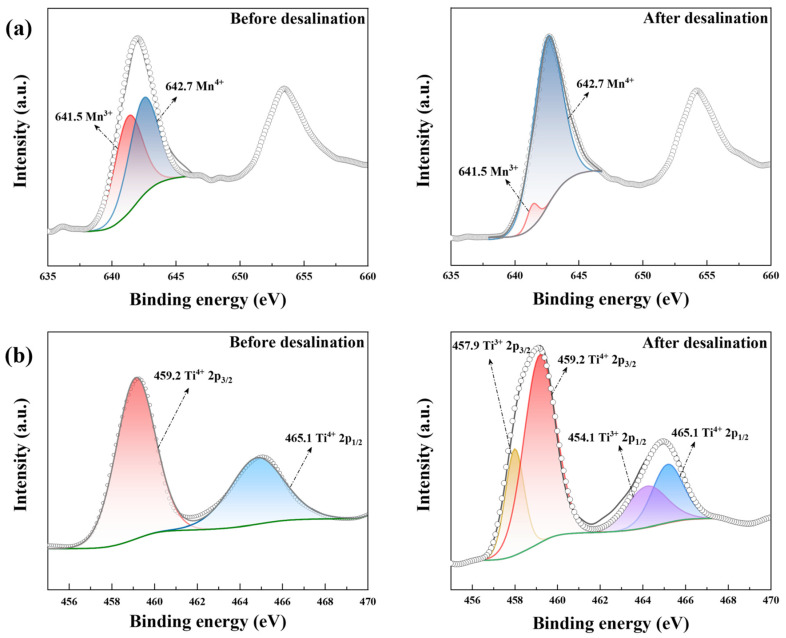
The XPS results of (**a**) Mn 2p in LiMn_2_O_4_/C and (**b**) Ti 2p in NaTi_2_(PO_4_)_3_/C before and after the desalination test at 1.8 V in 10 mM salt solution.

XRD (Figure 6) and XPS (Figure 7) tests were carried out to further study the variations of structures and valences in the electrode materials after desalination. The XRD peaks of LiMn_2_O_4_/C shifted rightwards after the deionization test, signifying the release of lithium ions during charging. However, there were no evident peak shifts for the anode after CDI, which was due to the tiny structure conversion of NaTi_2_(PO_4_)_3_/C during the electrochemical reaction, which resulted from the strong covalent bonds in the polyanion tetrahedrons of the crystal structures [49].

As shown in Figure 7a, the amount ratio of Mn^3+^ (641.5 eV) and Mn^4+^ (642.7 eV) [50] was approximately 1:1 in pristine LiMn_2_O_4_/C, which was a key characteristic of the LiMn_2_O_4_ material. After desalination, the amount of Mn^4+^ was increased to over 90%, indicating the intense oxidation of manganese ions within the charging process. As for NaTi_2_(PO_4_)_3_/C before desalination, the two diffraction peaks (459.2 eV and 465.1 eV) fitted well with Ti^4+^ 2p, demonstrating the valence of titanium in NaTi_2_(PO_4_)_3_/C was +4. After the CDI process, another two peaks showed up (Ti^3+^ 2p_1/2_: 464.1 eV, Ti^3+^ 2p_1/2_: 457.9 eV) [51], implying the presence of Mn^3+^. Thus, manganese and titanium were oxidated and reduced during the CDI process, respectively.

## 4. Conclusions

In conclusion, a novel dual-ion CDI system with lithium-ion battery cathode LiMn_2_O_4_/C and sodium-ion battery anode NaTi_2_(PO_4_)_3_/C was reported, which delivered an ultrahigh desalination capacity of 140.03 mg·g^−1^ with the initial salinity of 20 mM. With the low potential of the redox couple Ti^3+^/Ti^4+^ and the alleviated hydrogen evolution during CDI desalination, the voltage window of CDI in the new cell could be enlarged to 1.8 V. Besides, after coupling with NaTi_2_(PO_4_)_3_/C anode, the capacity of the LiMn_2_O_4_/C cathode was made full advantage of. The choice of the novel battery electrode couple resulted in an outstanding deionization performance, indicating a promising direction for future CDI desalination.

## Figures and Tables

**Table 1:** A comparison of the desalination performances of electrode couples in the literature and our work.

Electrode Couple	Charging Mode	Electric Intensity	Initial Salinity	CDI Capacity	Ref.
CNT||CNT	CV	1.2 V	3500 ppm	9.35 mg·g^−1^	[39]
porous carbon||porous carbon	CV	1.2 V	292 ppm	12.63 mg·g^−1^	[16]
MoS_2_||AC	CV	1.2 V	400 mM	8.81 mg·g^−1^	[40]
Na_4_Mn_9_O_18_||AC	CV	1.2 V	50 mM	31.2 mg·g^−1^	[20]
NaNiFe(CN)_6_||Na_2_NiFe(CN)_6_	constant current (CC)	0.28 mA·cm^−2^	20 mM	34 mg·g^−1^	[41]
Na_4_Ti_9_O_20_/C||AC	CV	1.4 V	1000 ppm	66.14 mg·g^−1^	[22]
Na_0.71_CoO_2_||Ag/rGO	CV	1.4 V	500 ppm	31 mg·g^−1^	[42]
Fe_4_[Fe(CN)_6_]_3_/rGO || rGO	CC	0.1 A·g^−1^	2500 ppm	80 mg·g^−1^	[43]
AC||Bi	CV	1.2 V	500 ppm	55.52 mg·g^−1^	[44]
Mo_1.33_C-MXene/CNT||Mo_1.33_C-MXene/CNT	CV	0.8 V	600 mM	15 mg·g^−1^	[45]
polypyrrole/C||polypyrrole/C	CV	1.2 V	500 ppm	34.03 mg·g^−1^	[46]
MnO_2_/C||MnO_2_/C	CV	1.2 V	500 ppm	30.86 mg·g^−1^	[47]
Na_3_V_2_(PO_4_)_3_/C||AgCl	CC	0.1 A·g^−1^	1000 ppm	98.0 mg·g^−1^	[48]
Ag||AgCl	CC	1 mA·cm^−2^	500 mM	85 mg·g^−1^	[22]
LiMn_2_O_4_/C||AC	CV	1.0 V	20 mM	117.3 mg·g^−1^	[25]
LiMn_2_O_4_/C||NaTi_2_(PO_4_)_3_/C	CV	1.8 V	20 mM	140.03 mg·g^−1^	this work

## Supplementary Materials

The following supporting information can be downloaded at: https://www.mdpi.com/article/10.3390/polym14214776/s1, Figure S1. (a) Cyclic voltammetry profiles of activated carbon with different scan ranges and (b) the galvanostatic charge-discharge (GCD) curve of activated carbon (−0.3~0.7 V versus Ag/AgCl) with a current density of 0.1 A·g^−1^ in 1.0 M NaCl solution; Figure S2. GCD curves of LiMn2O4/C (0.1 A·g^−1^) in (a) 1.0 M LiCl solution and (b) 1.0 M NaCl solution; Figure S3. The concentration variations of lithium ions near the cathode and chloride ions in the feed solution during CDI desalination at 1.8 V with the initial salt concentration of 10 mM.
