# Supplementary material for: A Novel Dual-Ion Capacitive Deionization System Design with Ultrahigh Desalination Performance
Source: Polymers (Basel). 2022 Nov 7;14(21):4776. doi: 10.3390/polym14214776 (PMC9657313; doi:10.3390/polym14214776)
Supplement: Supplementary file 1 [file polymers-14-04776-s001.zip › polymers-2010486-supplementary.pdf]

Supporting Information

A novel dual-ion capacitive deionization system design with ultrahigh desalination performance

Yuxin Jiang[1], Zhiguo Hou[2], Lvji Yan[1], Haiyin Gang[1], Haiying Wang[1,3,4,*] and Liyuan Chai[1,3,4]

1 School of Metallurgy and Environment, Central South University, Changsha 410083, China
2 School of Chemistry and Materials, University of Science and Technology of China, Hefei, 230026, China
3 Chinese National Engineering Research Center for Control and Treatment of Heavy Metal Pollution, Changsha 410083, China
4 Water Pollution Control Technology Key Lab of Hunan Province, Changsha 410083, China
* Correspondence: haiyw25@csu.edu.cn

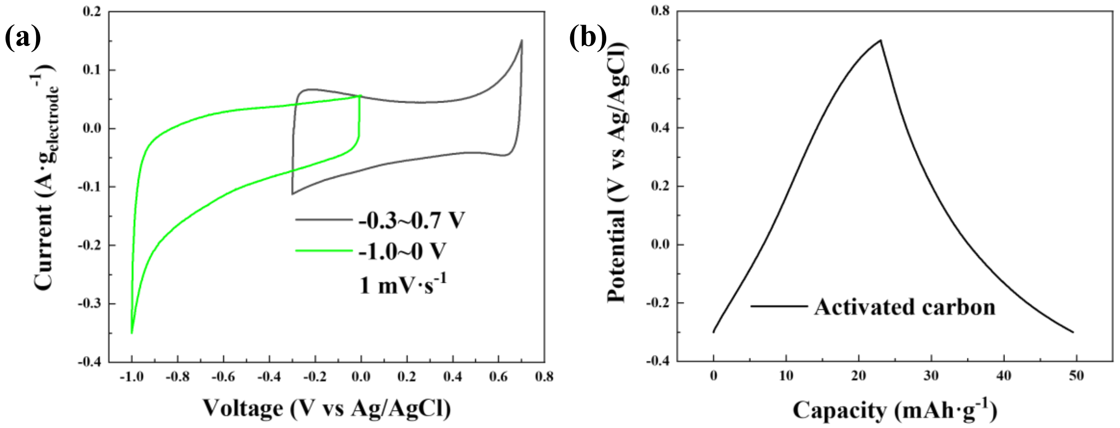

Figure S1. (a) Cyclic voltammetry profiles of activated carbon with different scan ranges and (b) the galvanostatic charge-discharge (GCD) curve of activated carbon (-0.3~0.7 V versus Ag/AgCl) with a current density of 0.1 A·g^{-1} in 1.0 M NaCl solution.

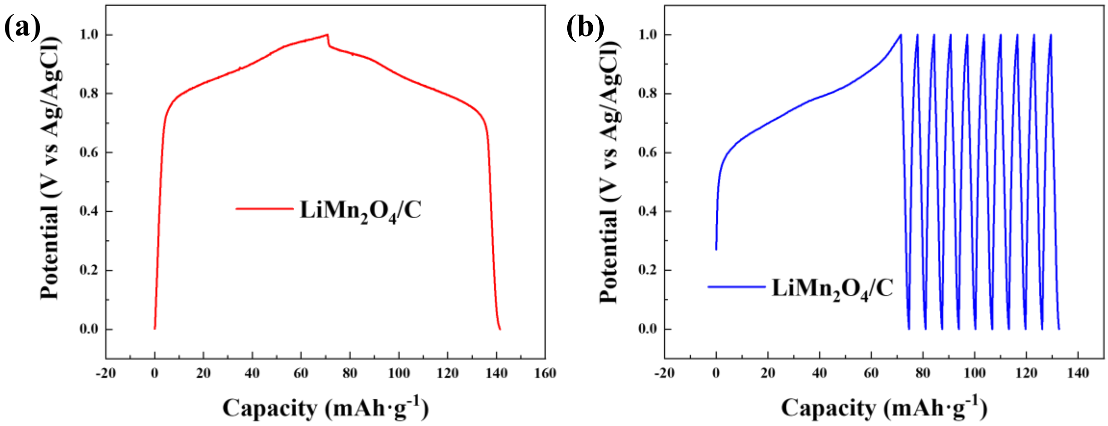

Figure S2. GCD curves of $LiMn_2O_4/C$ (0.1 A·g^{-1}) in (a) 1.0 M LiCl solution and (b) 1.0 M NaCl solution.

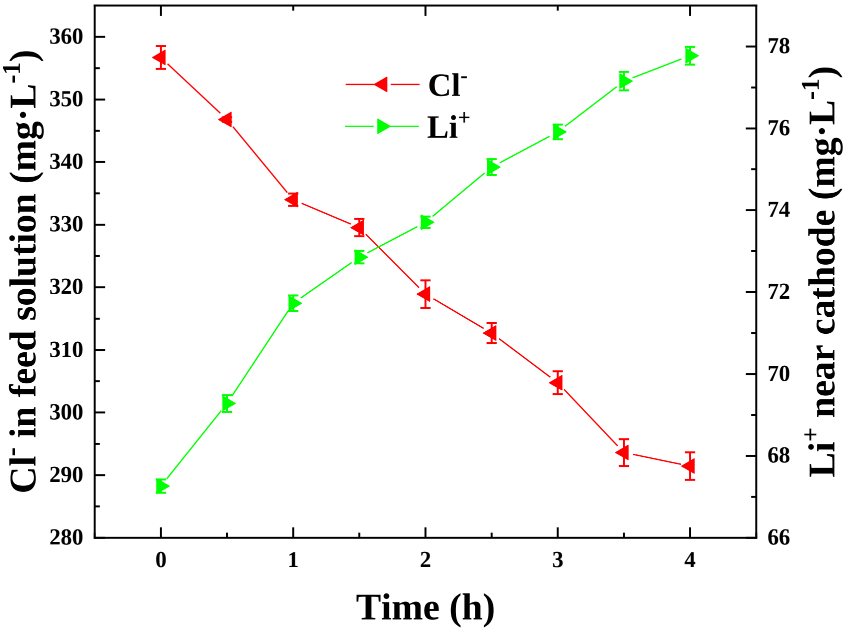

Figure S3. The concentration variations of lithium ions near the cathode and chloride ions in the feed solution during CDI desalination at 1.8 V with the initial salt concentration of 10 mM.
